# Insular biogeographic origins and high phylogenetic distinctiveness for a recently depleted lizard fauna from Christmas Island, Australia

**DOI:** 10.1098/rsbl.2017.0696

**Published:** 2018-06-13

**Authors:** Paul M. Oliver, Mozes P. K. Blom, Harold G. Cogger, Robert N. Fisher, Jonathan Q. Richmond, John C. Z. Woinarski

**Affiliations:** 1 Environmental Futures Research Institute, Griffith University, 170 Kessels Road, Nathan, Queensland 4111, Australia; 2 Biodiversity and Geosciences Program, Queensland Museum, South Brisbane, Queensland 4101, Australia; 3 Department of Bioinformatics and Genetics, Naturhistoriska Riksmuseet, Box 50007, 104 05 Stockholm, Sweden; 4 The Australian Museum, 1 William Street, Sydney, New South Wales, Australia; 5 U.S. Geological Survey, 4165 Spruance Road, Suite 200, San Diego, CA 92101, USA; 6 Threatened Species Recovery Hub, National Environmental Science Programme, Research Institute for the Environment and Livelihoods, Charles Darwin University, Casuarina, Darwin, NT 0810, Australia

**Keywords:** extinction, phylogenetic distinctiveness, Sunda Shelf, Wallace's Line, Wallacea

## Abstract

Striking faunal turnover across Asia and Australasia, most famously along the eastern edge of the Sunda Shelf or ‘Wallace's Line’, has been a focus of biogeographic research for over 150 years. Here, we investigate the origins of a highly threatened endemic lizard fauna (four species) on Christmas Island. Despite occurring less 350 km south of the Sunda Shelf, this fauna mostly comprises species from clades centred on the more distant regions of Wallacea, the Pacific and Australia (more than 1000 km east). The three most divergent lineages show Miocene (approx. 23–5 Ma) divergences from sampled relatives; and have recently become extinct or extinct in the wild, likely owing to the recent introduction of a southeast Asian snake (*Lycodon capucinus*). Insular distributions, deep phylogenetic divergence and recent decline suggest that rather than dispersal ability or recent origins, environmental and biotic barriers have impeded these lineages from diversifying on the continental Sunda Shelf, and thereby, reinforced faunal differentiation across Wallace's Line. Our new phylogenetically informed perspective further highlights the rapid loss of ancient lineages that has occurred on Christmas Island, and underlines how the evolutionary divergence and vulnerability of many island-associated lineages may continue to be underestimated.

## 1. Introduction

For over 150 years, biogeographers have noted marked biotic turnover between the Asian and Australian regions. This is most striking at the transition between the eastern edge of the Sunda Shelf and the islands of Wallacea (the Lesser Sundas and Sulawesi) [1], now widely referred to as Wallace's Line. This turnover has been linked to the former isolation and disjunct history of the Asian and Australian continental plates [2]. However, other factors such as biotic interactions (e.g. competition and predation) or environmental gradients may also underpin spatial variation in phylogenetic and evolutionary diversity [3,4]. For instance, downstream colonizations from the Sunda Shelf and into Australasia outnumber upstream colonizations [5,6], suggesting more species-rich and stable Sunda communities have been more resistant to biotic invasion.

Christmas Island is the westernmost oceanic island in the Australasian region. This isolated seamount (135 km^2^) lies over 1000 km west of mainland Australia and Wallacea, and 350 km south of Java in the Indian Ocean (electronic supplementary material, figure S1). Shortly after human settlement in the late 1880s, a diverse endemic fauna was documented [7], providing an exceptional baseline to study subsequent biotic change. While most endemic birds persist, the terrestrial reptile and mammal fauna have been severely depleted; four of five endemic mammals are extinct [8], as are three of the five reptiles (although two persist as *ex situ* captive colonies), with the reptile extinctions all occurring since 2009 [9]. Temporal and spatial patterns of decline of the reptile fauna strongly suggest the key cause was a primarily lizard eating snake (*Lycodon capucinus*) recently introduced from Asia, and also linked to extinctions of endemic lizard species in other islands in the Indian Ocean (see the electronic supplementary material, references). However, despite the isolation and conservation significance of this lizard fauna, its biogeographic origins remain unassessed.

Here, we show that four (of the five) endemic Christmas Island reptile species are nested within radiations centred on Wallacea, Australia or the Pacific (table 1). Phylogenetic and distributional data indicate the relevant clades have histories of persistence and/or dispersal on islands around the Sunda Shelf since the Miocene, but have been largely unable to colonize the shelf itself. Coupled with recent *Lycodon* driven extinctions, this suggests that biotic interactions or other environmental barriers, rather than dispersal ability, may underpin the contemporary rarity of these taxa on large landmasses west of Wallace's Line.

**Table 1.** Summary of biogeographic associations and divergence times for Christmas Island endemic lizards. Preferred model = combination molecular rate and speciation models with highest marginal likelihood. Ma, millions of years ago; HPD, highest posterior density; strict, strict clock rate model; lgnm, lognormal rate model; BD, birth–death speciation model; Yule, Yule speciation model.

	preferred models	sister lineage/nearest relatives	posterior support	location of nearest relative	mean split (Ma)	95% HPD (Ma)
*Cyrtodactylus sadleiri*combined datacombined no 3rds	lgnm + Yule	*Cyrtodactylus* sp. Bali	0.99	Bali	1	1–2
	lgnm + BD	*Cyrtodactylus* sp. Bali	0.71	Bali	2	1–3
*Lepidodactylus listeri*combined datacombined no 3rdsnuclear data only	strict + BD	*Lepidodactylus lugubris* group + *Luperosaurus joloensis*	0.77/07^a^	Philippines	23–26^a^	19–32^a^
	lgnm + BD	*Lepidodactylus lugubris* group	0.95	Philippines	26	21–31
	lgnm + BD	*Lepidodactylus lugubris* group	1.00	Philippines	28	22–34
*Emoia nativitatis*mtDNA	strict + Yule	*Emoia boettgeri*	1.00	Micronesia	13	9–18
*Cryptoblepharus egeriae*mtDNA	strict + Yule	*Cryptoblepharus metallicus* group	1.00	Australia	7	5–10

^a^Basal relationships poorly supported, posterior support and divergence estimates spanning two most closely related lineages shown.

## 2. Methods

We compiled sequence data for four (out of five) endemic reptile species from Christmas Island; two skinks *Cryptoblepharus egeriae* (Extinct in the Wild) and *Emoia nativitatis* (Extinct), and two geckos *Cyrtodactylus sadleiri* (Endangered) and *Lepidodactylus listeri* (Extinct in the Wild) (status from 2017 IUCN assessments). Tissues are not available for a very rare endemic blindsnake *Ramphotyphlops exocoeti* (Data Deficient) and from the Christmas Island population of the other native reptile, the non-endemic skink *Emoia atrocostata*, whose Christmas Island population was recently extirpated. For each genus, alignments included recognized species and divergent but taxonomically unrecognized lineages, mostly from published studies [10–12] (electronic supplementary material, tables S1–S4). Dated phylogenies were estimated using Bayesian approaches [13] run for 20 000 000 generations, sampling every 20 000, with the first 20% of trees discarded as burn-in.

Gecko analyses used secondary age priors for intrageneric nodes (electronic supplementary material, table S2) derived from a fossil-calibrated five nuclear gene alignment spanning gekkotans (electronic supplementary material, figure S2), and were run on combined datasets with, and without, third codons. One gecko lineage (*Lepidodactylus*) showed a very deep split from other sampled taxa, so additional nuclear gene analyses were run to assess robustness of age inference. A reliable and dated overall family level tree for skinks is not available [14], so we combined (a) broad mitochondrial rate-based calibrations and (b) age priors from a rate-smoothed chronogram for the relevant clade (the *Eugongylus* group) (electronic supplementary material, methods). Alignments and .xml files are available online [15].

## 3. Results and discussion

**Figure 1.**
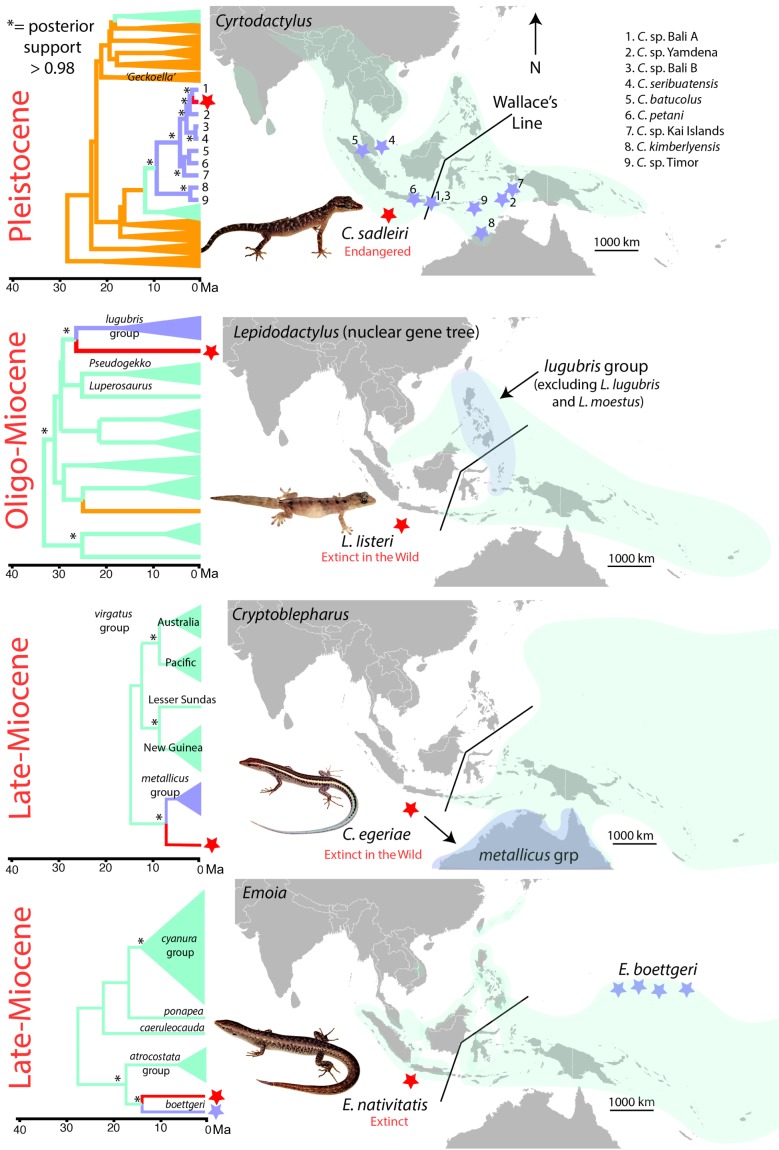
Biogeographic relationships and temporal divergence of endemic Christmas Island lizards. Taxa from Christmas Island (red star); sister lineages or closest relatives (light blue); overall natural distribution of congeners (light green). Lineages from the Sunda Shelf or mainland Asia are orange.

All Christmas Island endemic lizards have biogeographic affinities with radiations centred to the east of Wallace's Line (figure 1). The three extinct, or extinct in the wild, species are most genetically divergent (approx. 5–25 Myr separation from closest sampled relatives) and geographically disjunct, associating with lineages centred in mainland Australia (*Cryptoblepharus;* approx. 1500 km), The Philippines (*Lepidodactylus*; approx. 2500 km) and Micronesia (*Emoia*; approx. 4000 km). One species still extant in the wild, *Cyrtodactylus sadleiri*, is less divergent, and closely related to taxonomically unassigned populations from Bali (approx. 1000 km east) and Yamdena (approx. 3000 km east). Genetic divergences at the *ND2* locus between these lineages (0.043–0.063% pairwise uncorrected) are low for gecko species [16]. These lineages sit within a broader late Miocene radiation centred on Wallacea, from which several dispersals westwards onto islands and other coastal or savannah habitats around the edge of the Sunda Shelf are inferred [17].

As-yet-unsampled taxa could split key branches in our phylogenies and reduce the phylogenetic distinctiveness that we report for the Christmas Island endemics (electronic supplementary material, further discussion). However, very few taxa in these relevant clades are currently known from the Sunda Shelf, so the broad pattern that all Christmas Island endemics are derived from clades centred east of Wallace's Line is likely to be robust. A converse possibility that one or more of the Christmas Island taxa have anthropogenic origins seems unlikely: none is human commensal; divergences pre-date anthropogenic movement across Wallacea, and all were collected prior to or within 10 years of the island's original settlement [7].

All four lizard radiations with endemic taxa on Christmas Island have a long history in regions east (Wallacea) and south (Christmas Island and/or Australia) of the Sunda Shelf, and show evident overwater dispersal ability (three of the genera also have naturally dispersed thousands of kilometres eastwards into the Pacific, or westwards to the Malagasy region [18]). It is, therefore, striking that they are only marginally present on the Sunda Shelf (absent from rainforest, and restricted to coastal or savannah habitats) [19]. The introduction (in the 1980s) of a predatory genus (*Lycodon*) widespread on the Sunda Shelf is also presumed to have caused the recent loss of the three most phylogenetically divergent Christmas Island taxa [9], as well as the Christmas Island populations of the widespread insular lizard *Emoia atrocostata.* Other insular associated lizard [20] and bird [4] radiations that are widespread in Wallacea also show distributional boundaries along the eastern edge of the Sunda Shelf, while ranging widely into Melanesia and the Pacific. Insular distributions and biotic vulnerability are often linked to the loss of key traits subsequent to colonization of simple, isolated and benign island communities (e.g. flightlessness) [21]. Our results suggest a converse idea that along the edge of the Sunda Shelf or ‘Wallace's Line’ sharp distributional turnover linked to dispersal limitations in continental taxa [1,4] may be further reinforced by an inability of island-associated radiations to successfully penetrate (or persist in) complex continental systems.

Phylogenetic analyses continue to reveal divergent endemic lineages on other relatively small islands across the Indo-Pacific [12], and elsewhere [22]. The study of such highly isolated biotas can provide an important perspective on the underlying mechanisms that explain contemporary biogeographic distributions. However, unfortunately, while—by definition—many island relicts have persisted over very long periods, many fare poorly when new competitors and predators are anthropogenically introduced [21]. In such cases, millions of years of unique evolutionary history may be lost with extraordinary rapidity. Christmas Island provides an unusually well-documented case study, and we estimate that the three recently extinct species (including the two extinct in the wild) each represented millions of years of unique evolutionary history. Therefore, maintenance and enhancement of conservation management of Christmas Island's two endemic lizard species now occurring only as captive populations should remain a priority [23]. More broadly, it seems likely that similar declines or extinctions on islands across the Indian Ocean and Pacific may have gone unrecorded, and further, that other similarly divergent and vulnerable taxa may remain extant, but undocumented. We suggest that there is a priority research need to document endemic island faunas, to understand their evolutionary significance and phylogenetic distinctiveness, and to try to enforce better biosecurity protocols to halt the ongoing spread of *L. capucinus* [24] and other taxa [25] that could devastate some of their most evolutionarily distinctive endemic species.

## Supplementary Material

Oliver et al. 2017 Supplementary Files

## Data accessibility

Specimen details are provided in the electronic supplementary material. All supplementary material and alignments are available on Dryad (http://dx.doi.org/10.5061/dryad.s66p0) [15].
