# Supplementary material for: Insular biogeographic origins and high phylogenetic distinctiveness for a recently depleted lizard fauna from Christmas Island, Australia
Source: Biol Lett. 2018 Jun 13;14(6):20170696. doi: 10.1098/rsbl.2017.0696 (PMC6030605; doi:10.1098/rsbl.2017.0696)
Supplement: Oliver et al. 2017 Supplementary Files [file rsbl20170696supp1.docx]

**Electronic Supplementary Material for:**

Insular biogeographic origins and high phylogenetic distinctiveness for a recently depleted lizard fauna from Christmas Island, Australia

Paul M. Oliver, Mozes P. K. Blom, Harold G. Cogger, Robert N. Fisher, Jonathan Q. Richmond and John C.Z. Woinarski

**Supplementary methods and discussion**

**Additional details of sample selection, nucleotide processing and analysis**

Alignments consisted of one mitochondrial locus (ND2) with varying amounts of flanking tRNA, in addition to up three nuclear loci. Sequence data were aligned and checked for frameshift mutations and translations. Partitioning strategies for geckos were selected using Partitionfinder v.2 [28]. For *Cyrtodactylus* partitions and models were: 1 mtDNA firsts, seconds and tRNA (GTR+G); 2 mtDNA thirds (GTR+G); 3 nuclear DNA (GTR+G). For *Lepidodactylus* partitions and models were: 1 mtDNA firsts; 2 mtDNA seconds; 3 mtDNA thirds; 4 nuclear firsts; 5 nuclear seconds; 6 nuclear thirds (all partitions with GTR+G). For skinks we used molecular rate calibrations (see below) so the mitochondrial data was run as a single partition (as most available general mtDNA rates are not calibrated by codon). After Bayesian analyses adequate effective samples sizes (ESS) were confirmed using Tracer v1.6.0 [29].

All dating analyses were run using 4 different combinations of rate (strict clock, uncorrelated lognormal) and speciation (Yule, birth-death) models and compared using highest marginal likelihoods implemented in Tracer [29]. Running multiple analyses also allowed us to determine consistency of age estimates across different sets of assumptions.

**Details of dating priors**

**Geckos**

For initial estimation of the crown-radiation ages of *Lepidodactylus* and *Cyrtodactylus* we used a published dataset comprising five nuclear genes and including almost all recognised gecko genera, and multiple examplars of taxa spanning the diversity of these two focal genera [15]. For this analysis the following settings were left the same as per the original analysis in which they were presented: nuclear data run as a single partition, Yule speciation prior, uncorrelated lognormal model of molecular rate evolution, and four fossil calibrations spanning Squamata (Figure S2). However two younger fossil and biogeographic calibrations, that have been shown to be of questionable utility as dating constraints were removed [30,31]. Age priors from the resultant tree (figure S2) were then harvested to apply as normally distributed constraints on downstream analysis of *Cyrtodactylus* and *Lepidodactylus*.

For *Cyrtodactylus* of the four different combination of rate and speciation models used, the combining lognormal and Birth-Death speciation prior had the highest marginal likelihood (as determined by AICM implemented in Tracer v 1.6 [29]). However crown age estimates varied very little across different combinations of data and priors (e.g mean split for *Cyrtodactylus sadleiri* from *Cyrtodactylus* sp. Bali was always estimated as Pleistocene: <2 mya). Investigation of dated trees suggest that posterior estimates for basal divergences (between a Myanmar clade and the remainder of taxa were underestimated (e.g preferred tree with no third codons, lognormal and Birth Death priors: posterior 95%HPD 19.7 – 34.4 mya vs. prior mean 31 mya SD 5.0). However, the second shallower calibration we applied to node containing most south-east Asian and Melanesian *Cyrtodactylus* was broadly overlapping across prior and posterior (posterior 95% Highest Posterior Density[HPD] 14.6–26.9 vs prior mean 21.0 SD 4.0). As we were more interested in shallower splits, we left crown ages underestimated, in preference to experimenting with alternative models that would be likely to inflate focal tip ages (i.e a tighter constraint on the crown age or deeper calibrations)(figure S3).

For the combined dataset of *Lepidodactylus* the uncorrelated lognormal and Birth-Death speciation prior had the highest marginal likelihood. Age estimates were again consistent across data combinations, model and rate combinations (means 23–26mya) (figure S4). Age estimates for *Lepidodactylus* based on the combined datasets were far older than any other Christmas Island endemic. To further investigate for any potential effect of mitochondrial saturation to inflate ages in such deeply divergent taxa we ran an additional set of analyses for this genus focusing a nuclear gene only alignment (figure S4). All settings for the nuclear loci in this analysis were as per the combined dataset.

**Skinks**

No large genus level comprehensive nuclear gene and fossil calibrated phylogeny has been produced specifically for skinks. Published and dated tree-of-life frameworks (macrophylogenies) do include a large number of skinks[32], however dating analyses based on these datasets produce inconsistent date estimates[16]. Furthermore ages for skink radiations estimated from macrophylogenies are also inconsistent with smaller and more focused fossil calibrated studies focusing on key components of the Australia fauna[5,33]. One key driver of these discrepancies may be the combination of heavily saturated mitochondrial data and deep old calibrations in dating analyses based on the macrophylogenies [34]. In light of these issues, to initially estimate the ages of key divergence events within the two focal genera we subsampled the relevant clade (Eugongylus group plus *Lygosoma* and *Lamprolepis* taxa as outgroups) from a large-scale concatenated phylogeny for all of Squamata [35]. We then estimated timeframes of evolution across this tree used penalised rate-smoothing as implemented in r8s v. 1.7 [36]. Mean age priors for the four deepest nodes in this tree (all highlighted in figure S5), were taken from a recent nuclear gene, fossil calibrated analysis of the evolutionary timeframes for Australian skinks [33]. Confidence limits for node ages were estimated by varying the smoothing parameter in 12 increments between 0.1-20 to generate 12 independent trees, that were then combined into a consensus. The resultant tree contained age estimates for the divergence between key taxa nested within both *Emoia* and *Cryptoblepharus,* and for which we had mitochondrial sampling*.* These were used to provide relatively tight uniformly distributed age priors for relevant nodes in individual BEAST analyses for each genus (Table SI2, figures S6–S7). In each of these analyses we further placed a broad rate prior on the (unpartitioned) mitochondrial gene alignment of between 1–4% pairwise per million years – reflecting a range of estimates published for the rate of evolution of ND2 [37,38].

For both *Cryptoblepharus* and *Emoia*, of the four different combinations of rate and speciation priors used, strict-clock with Yule speciation prior had the highest marginal likelihood. For both skink taxa all dating and phylogenetic analyses (figures S6–7) were also compared against nuclear gene/exon capture datasets for the relevant genera that are published [11] or in prep (JQR, RNF), and against overall timeframes for skink evolution that have been published elsewhere [33]. Nonetheless we emphasise that these dates derived from secondary calibration strategies combined with saturated mitochondrial data should be regarded with corresponding caution. Hence throughout this paper we only focus on broad timeframes of inference.

**A comment on coalescent dating approaches**

We did not use coalescent approaches to estimate divergences ages as: a) only single loci were available for skink taxa, and b) and given the deep and very shallow divergence of the two gecko taxa for which we had in multigene trees, inflation of age estimates in concatenated datasets (the key problem with inference of dates from concatenated datasets) is very unlikely to change the broad biogeographic and temporal inferences we draw here[39].

The estimated timing of divergence from nearest sampled relatives varies from Pleistocene (*Cyrtodactylus*), mid- to late Miocene (*Cryptoblepharus* and *Emoia*) to early Miocene (*Lepidodactylus*) (Table 1, figure 1). These date estimates were highly consistent across four different combinations speciation and molecular rate priors, and across datasets including varying amounts of mitochondrial data.

**Missing taxa and taxonomic considerations**

An important potential caveat to the biogeographic interpretations we present here is that as yet unsampled taxa could split key branches in our phylogenies, reducing our estimates for phylogenetic divergence or altering geographic patterns. We discuss this potential by genus below.

*Cyrtodactylus*

Many unsampled taxa (both recognised and unrecognised) in this exceptionally diverse genus occur on the Sunda Shelf, the Lesser Sundas and elsewhere in Wallacea. However, in light of the shallow nature of splits between *Cyrtodactylus sadleiri* and *Cyrtodactylus* sp. Bali (and for that matter *C*. sp Yamdena), the immediate priority would be to establish whether these taxa are conspecific. Therefore discovery of additional taxa or populations is not likely to change our interpretation that *C. sadleiri* has colonised Christmas Island relatively recently.

More sampling may reveal additional closely related taxa/populations on the Sunda Shelf, potentially altering our interpretation that *Cyrtodactylus sadleiri* is part of a clade centred on Wallacea, that has more recently moved westwards into open/disturbed/insular habitat around the edges of the Sunda shelf. One other genetically sample taxa we have from Java (*Cyrtodactylus* ‘*marmoratus*’ from Cibodas, ABTC48078) from [10] is not closely related to the Wallacean *Cyrtodactylus* radiation in which *C. sadleiri* sits. In contrast *Cyrtodactylus petani* is deeply nested with the Wallacean *Cyrtodactylu*s radiation, suggesting that it has secondarily colonised open and highly disturbed habitats in Java [40].

*Lepidodactylus*

This genus includes numerous species of small, rare and relatively cryptic taxa, many of which also show evidence of very deep evolutionary divergences [41]. There is a high probability that populations or species of *Lepidodactylus* from Indonesia are allied to *Lepidodactylus listeri*. We only present comparisons against described species below, but emphasise that as yet undescribed taxa may also be involved.

*Lepidodactylus listeri* has undivided lamellae (often considered a pleisomorphic trait in this group) [42]. The most geographically proximate genetically unsampled taxa from the Lesser Sunda Islands (*Lepidodactylus lomboensis* and *Lepidodactylus intermedius*), both have divided subdigital lamellae, suggesting they are distantly related. Further east *Lepidoactylus oorti* is a very rare species known only from Tanimbar, Tëun and Serua Islands in eastern Maluku. This species is similar in colour pattern and lamellae structure to *L. listeri*, although it is larger (SVL >50 vs < 50mm) and has a much higher number of pores (28-29 vs. 9-13) [42]. While this morphologically differentiation suggest these two taxa are unlikely to be conspecific, a close phylogenetic relationship is possible. Other taxa show evidence of dispersal between Christmas Island and Maluku including *Cyrtodactylus* (this study). A relationship between these taxa would change the temporal but not overall biogeographic conclusions presented above. *Lepidodactylus oorti* has also not been seen since its original description, notwithstanding a number of herpetological surveys around Tanimbar. Two of three known records are from small and geologically unstable volcanic islands, fitting with the hypothesis that this taxon is highly dispersive, but often associated with poor marginal habitats that have fewer competitors and predators.

*Cryptoblepharus*

*Cryptoblepharus egeriae* from Christmas Island is highly distinct in morphology and differs from all other congeners in the Lesser Sundas and Maluku (*C. baliensis*, *C. burdeni*, *C. cursor*, *C. intermedius*, *C. keiensis*, *C. leschnault*, *C. renschi* and *C. schlegelianus* in having a) 8 supracilary scales, b) unfused interparietal and frontoparietal scales and c) a bright blue tail [43]. The following taxa from the Lesser Sundas/Maluku have also been included in genetic analyses here (*C. lecschenault*) or not yet published (*C. burdeni*, *C. cursor*, *C. keiensis*, *C. intermedius*, *C. renschi*; Blom et al. in prep) and none are closely related to *Cryptoblepharus egeriae*.

*Emoia*

*Emoia nativitatis* was placed in the *atrocostata* group on the basis of suite of shared morphological characters including a) alpha type palate, b) fused nasal bones and c) rounded subdigital lamellae [44]. Subsequent molecular phylogenetic analyses (here, and Richmond et al. in prep) have supported the monophyly and distinctiveness of this morphologically defined group from all other recognised species of *Emoia* (Richmond et al. in prep). The Lesser Sundas and Maluku are home to relatively few *Emoia*, and all are very different morphologically, and all but *Emoia atrocostata* also do not occur west of Wallacea [45].

No tissue samples are available for the recently extinct Christmas Island population of *Emoia atrocostata*. While a name is available for this form (*Emoia sinus*, Smith 1929), it is impossible to assess its distinctiveness based on available information. On the one hand small and apparently isolated and ephemeral populations of *Emoia atrocostata* have been reported from scattered island around Indonesia, including Krakatau off the coast of Java [46] and Bali [47]. These data suggest this species does have the ability to regularly disperse long distances of overwater. Conversely phylogenetic data (Richmond et al. in prep) suggest deep structure within this species. In the absence of tissues it is difficult to assess the evolutionary divergence of this now extinct form from other populations of *Emoia atrocostata*. This clearly highlights the value and importance of tissue collections, even for taxa that may initially be common and widespread.

28. Lanfear R, Calcott B, Ho SYW, Guindon S. 2012 PartitionFinder: Combined selection of partitioning schemes and substitution models for phylogenetic analyses. *Mol. Biol. Evol.* **29**, 1695–1701. (doi:10.1093/molbev/mss020)

29. Rambaut A, Suchard MA, Xie D, Drummond AJ. 2014 Tracer v1.6.

30. Renner SS. 2016 Available data point to a 4-km-high Tibetan Plateau by 40 Ma, but 100 molecular-clock papers have linked supposed recent uplift to young node ages. *J. Biogeogr.* **43**, 1479–1487. (doi:10.1111/jbi.12755)

31. Lee MSY, Oliver PM, Hutchinson MN. 2009 Phylogenetic uncertainty and molecular clock calibrations: A case study of legless lizards (Pygopodidae, Gekkota). *Mol. Phylogenet. Evol.* **50**, 661–666. (doi:10.1016/j.ympev.2008.11.024)

32. Zheng Y, Wiens JJ. 2015 Combining phylogenomic and supermatrix approaches, and a time-calibrated phylogeny for squamate reptiles (lizards and snakes) based on 52 genes and 4162 species. *Mol. Phylogenet. Evol.* (doi:10.1016/j.ympev.2015.10.009)

33. Skinner A, Hugall AF, Hutchinson MN. 2011 Lygosomine phylogeny and the origins of Australian scincid lizards. *J. Biogeogr.* **38**, 1044–1058. (doi:10.1111/j.1365-2699.2010.02471.x)

34. Brandley MC, Wang Y, Guo X, Montes De Oca AN, Fería-Ortíz M, Hikida T, Ota H. 2011 Accommodating heterogenous rates of evolution in molecular divergence dating methods: An example using intercontinental dispersal of plestiodon (Eumeces) lizards. *Syst. Biol.* **60**, 3–15. (doi:10.1093/sysbio/syq045)

35. Pyron RA, Burbrink FT, Wiens JJ. 2013 A phylogeny and revised classification of Squamata, including 4161 species of lizards and snakes. *BMC Evol. Biol.* **13**, 1. (doi:10.1186/1471-2148-13-93)

36. Sanderson MJ. 2003 Evolution and divergence times in the absence of a molecular Clock. *Evolution (N. Y).* **19**, 301–302. (doi:10.1093/bioinformatics/19.2.301)

37. Eo SH, DeWoody JA. 2010 Evolutionary rates of mitochondrial genomes correspond to diversification rates and to contemporary species richness in birds and reptiles. *Proc. R. Soc. London Biol. Sci.* **277**, 3587–3592. (doi:10.1098/rspb.2010.0965)

38. Oliver PM, Adams M, Doughty P. 2010 Molecular evidence for ten species and Oligo-Miocene vicariance within a nominal Australian gecko species (*Crenadactylus ocellatus*, Diplodactylidae). *BMC Evol. Biol.* **10**, 386. (doi:10.1186/1471-2148-10-386)

39. Ogilvie HA, Heled J, Xie D, Drummond AJ. 2016 Computational performance and statistical accuracy of∗BEAST and comparisons with other methods. *Syst. Biol.* **65**, 381–396. (doi:10.1093/sysbio/syv118)

40. Riyanto A, Grismer LL, Wood PL. 2015 The fourth Bent-toed Gecko of the genus *Cyrtodactylus* (Squamata: Gekkonidae) from Java, Indonesia. *Zootaxa* **4059**, 351–363. (doi:10.11646/zootaxa.4059.2.6)

41. Oliver PM, Brown RM, Kraus F, Rittmeyer E, Travers SL, Siler CD, Oliver PM. 2018 Lizards of the lost arcs: mid-Cenozoic diversification , persistence and ecological marginalization in the West Pacific. *Proc. R. Soc. B* **285**, 1–9.

42. Brown WC, Parker F. 1977 Lizards of the Genus *Lepidodactylus* (Gekkonidae) From the Indo-Australian Archipelago and the Islands of the Pacific, With Descriptions of New Species. *Proc. Calif. Acad. Sci.* **41**, 253–265.

43. Horner P. 2007 Systematics of the snake-eyed skinks, *Cryptoblepharus* Wiegmann (Reptilia: Squamata: Scincidae) - an Australian-based review. *Beagle Rec. Museums Art Gall. North. Territ. Suppl.* **3**, 21–198.

44. Brown WC. 1991 Lizards of the genus *Emoia* (Scincidae) with observations on their evolution and biogeography. *Mem. Calif. Acad. Sci.* **15**, 1–94.

45. How RA, Durrant B, Smith LA, Saleh N. 1998 *Emoia* (Reptilia: Scincidae) from the Banda Arc islands of eastern Indonesia: variation in morphology and description of a new species. *Rec. West. Aust. Museum* **19**, 131–139.

46. Thornton I. 1996 *Kratatau: The Destruction and Reassembly of an Island Ecosystem*. Cambridge: Harvard University Press.

47. Somaweera R. 2017 *A Naturalist’s Guide to the Reptiles & Amphibians of bali*. Oxford: John Beaufoy Publishing Ltd.

**Additional key references for *Lycodon capuchinus* biology and impact.**

Cheke AS. (1987) An ecological history of the Mascarene Islands, with particular reference to extinctions and introductions of land vertebrates. In 'Studies of Mascarene Island birds'. (Ed. AW Diamond) pp. 5-89. Cambridge University Press: Cambridge

Jackson K, Fritts TH. (2004). Dentitional specialisations for durophagy in the Common Wolf snake, *Lycodon aulicus capucinus. Amphibia-Reptilia* **25**, 247-254.

Rodda GH, Fritts TH, Campbell EWI, Dean-Bradley K, Perry G, Qualls CP (2002) Practical concerns in the eradication of island snakes. In 'Turning the tide: the eradication of invasive species'. (Eds CR Veitch and MN Clout) pp. 260-265. IUCN SSC Invasive Species Specialist Group: Gland, Switzerland and Cambridge

Table S1. Details of *Cyrtodactylus* samples included in analyses. Mainly from Wood et al. 2012. New sequenced samples of *Cyrtodactylus sadleiri* are all from specimens lodged in the Australian Museum: AMSR152680 - MH105036; AMSR152675 - MH105037; AMS152676 - AMSR152675.

| ***Species*** | **ND2** | **MXRA5** | **PDC** | **RAG1** |
| --- | --- | --- | --- | --- |
| *Cyrtodactylus 'marmoratus'/halmahericus* | JX440546 | JX440604 | JX440656 | JX440706 |
| *Cyrtodactylus "Gobe Ridge"* | JQ820322 |  |  |  |
| *Cyrtodactylus (Geckoella) deccanensis* | JX440521 |  | JX440630 | JX440681 |
| *Cyrtodactylus (Geckoella) triedra* | JX440522 | JX440578 | JX440631 | JX440682 |
| *Cyrtodactylus adorus* | HQ401166 |  |  |  |
| *Cyrtodactylus agusanensis* | GU550818 |  |  |  |
| *Cyrtodactylus angularis* | JX440523 | JX440579 | JX440632 | JQ945301 |
| *Cyrtodactylus annandalei* | JX440524 |  | JX440633 | JX440683 |
| *Cyrtodactylus annulatus* | GU366085 |  |  |  |
| *Cyrtodactylus arcanus* | JQ820319 |  |  |  |
| *Cyrtodactylus astrum* | JX519472 |  |  |  |
| *Cyrtodactylus aurensis* | JX440525 |  | JX440580 | JX440684 |
| *Cyrtodactylus australotitiwangsaensis* | JX519484 |  |  |  |
| *Cyrtodactylus ayeyarwadyensis* | JX440526 | JX440581 | JX440634 | JX440685 |
| *Cyrtodactylus baluensis* | GU366080 |  |  |  |
| *Cyrtodactylus batucolus* | JQ889178 | JX440582 | JX440635 | JX440686 |
| *Cyrtodactylus bintangrendah* | JX519487 |  |  |  |
| *Cyrtodactylus biordinis* | MF673951 |  | MF673976 | MF673969 |
| *Cyrtodactylus boreoclivus* | JQ820319 |  |  |  |
| *Cyrtodactylus boreoclivus* | JQ820307 |  |  |  |
| *Cyrtodactylus boreoclivus* | JQ820308 |  |  |  |
| *Cyrtodactylus brevidactylus* | JX440527 | JX440583 | JX440636 | JX440687 |
| *Cyrtodactylus capreoloides* | JQ820312 |  |  |  |
| *Cyrtodactylus capreoloides* | JQ820313 |  |  |  |
| *Cyrtodactylus capreoloides* | JQ820311 |  |  |  |
| *Cyrtodactylus capreoloides* | MF706374 |  |  |  |
| *Cyrtodactylus capreoloides* | MF706375 |  |  |  |
| *Cyrtodactylus cavernicolus* | MF706373 |  |  |  |
| *Cyrtodactylus cf. condorensis* | JX440531 | JX440586 | JX440639 | JX440690 |
| *Cyrtodactylus chanhomeae* | JX440529 | JX440584 | JX440637 | JX440688 |
| *Cyrtodactylus chrysopylos* | JX440530 | JX440585 | JX440638 | JX440689 |
| *Cyrtodactylus condorensis* | KT013196 |  |  |  |
| *Cyrtodactylus consobrinus* | JX440532 | JX440587 | JX440640 | JX440691 |
| *Cyrtodactylus darmandvillei* | JX440533 | JX440588 | JX440641 | JX440692 |
| *Cyrtodactylus darmandvillei* | KU232615 |  |  |  |
| *Cyrtodactylus darmandvillei* | KU232616 |  |  |  |
| *Cyrtodactylus darmandvillei* | JX440533 |  |  |  |
| *Cyrtodactylus darmandvillei* | KU232618 |  |  |  |
| *Cyrtodactylus dati* | KT013104 |  |  |  |
| *Cyrtodactylus eisenmanae* | JX440534 | JX440589 | JX440642 | JX440693 |
| *Cyrtodactylus elok* | JQ889180 | JX440590 | JX440643 | JX440694 |
| *Cyrtodactylus epiroticus* | JX440535 | JX440591 | JX440644 | JX440695 |
| *Cyrtodactylus equestris* | KT835457 |  |  |  |
| *Cyrtodactylus fasciolatus* |  |  | HM622366 | HM622351 |
| *Cyrtodactylus feae* | JX440536 | JX440592 | JX440645 | JX440696 |
| *Cyrtodactylus gansi* | JX440537 | JX440593 | JX440646 | JX440697 |
| *Cyrtodactylus grismeri* | JX440538 | JX440594 | JX440647 | JX440698 |
| *Cyrtodactylus guakanthanensis* | KU253576 |  |  |  |
| *Cyrtodactylus gunungsenyumensis* | KU253585 |  |  |  |
| *Cyrtodactylus hidupselamanya* | KX011412 |  |  |  |
| *Cyrtodactylus hontreensis* | JX440539 | JX440595 |  | JX440699 |
| *Cyrtodactylus hoskini* | HQ401119 |  |  |  |
| *Cyrtodactylus interdigitalis* | JQ889181 | JX440596 | JX440648 | JX440700 |
| *Cyrtodactylus intermedius* | JQ889182 | JX440597 | JX440649 | JX440701 |
| *Cyrtodactylus intermedius* | KT013107 |  |  |  |
| *Cyrtodactylus irregularis* | JX440540 | JX440598 | JX440650 | JQ945302 |
| *Cyrtodactylus irregularis* | KT013167 |  |  |  |
| *Cyrtodactylus jambangan* | GU366100 |  |  |  |
| *Cyrtodactylus jarujini* | JX440541 | JX440599 | JX440651 | JQ945303 |
| *Cyrtodactylus jellesmae* | JX440542 | JX440600 | JX440652 | JX440702 |
| *Cyrtodactylus khasiensis* | JX440543 |  |  |  |
| *Cyrtodactylus kimberleyensis* | JX440544 | JX440601 | JX440653 | JX440703 |
| *Cyrtodactylus klugei* | HQ401198 |  |  |  |
| *Cyrtodactylus langkawiensis* | JX519495 |  |  |  |
| *Cyrtodactylus leegrismeri* | KT013195 |  |  |  |
| *Cyrtodactylus lekaguli* | KX011425 |  |  |  |
| *Cyrtodactylus loriae* | EU268350 | JX440602 | EU268319 | EU268289 |
| *Cyrtodactylus loriae* | EU268350 |  |  |  |
| *Cyrtodactylus loriae 2* | KM086127 |  |  |  |
| *Cyrtodactylus louisiadensis* | HQ401190 |  |  |  |
| *Cyrtodactylus louisiadensis* | HQ401190 |  |  |  |
| *Cyrtodactylus macrotuberculatus* | JX440545 | JX440603 | JX440654 | JX440704 |
| *Cyrtodactylus malayanus* |  |  | JX440655 | JX440705 |
| *Cyrtodactylus malayanus* | GU550732 |  |  |  |
| *Cyrtodactylus marmoratus* | GQ257747 |  |  |  |
| *Cyrtodactylus mcdonaldi* | HQ401150 |  |  |  |
| *Cyrtodactylus medioclivus* | JQ820294 |  |  |  |
| *Cyrtodactylus medioclivus* | JQ820295 |  |  |  |
| *Cyrtodactylus metropolis* | KU253578 |  |  |  |
| *Cyrtodactylus mimikanus* | JQ820316 |  |  |  |
| *Cyrtodactylus minor* | JQ820318 |  |  |  |
| *Cyrtodactylus murua* | KT363953 |  |  |  |
| *Cyrtodactylus novaeguineae 1* | FK8669 |  |  |  |
| *Cyrtodactylus novaeguineae 2* | JX440547 | JX440605 | HQ426185 | HQ426274 |
| *Cyrtodactylus novaeguineae 3* | JQ820297 |  |  |  |
| *Cyrtodactylus oldhami* | JX440548 | JX440606 | JX440657 | JX440707 |
| *Cyrtodactylus pantiensis* | JQ889185 | JX440607 | JX440658 | JX440708 |
| *Cyrtodactylus papuensis* | JQ820321 |  |  |  |
| *Cyrtodactylus papuensis west* | JQ830315 |  |  |  |
| *Cyrtodactylus paradoxus* | JX440549 | JX440608 | JX440659 | JX440709 |
| *Cyrtodactylus payacola* | JQ889190 |  |  |  |
| *Cyrtodactylus peguensis* | GU550727 |  |  |  |
| *Cyrtodactylus peguensis* | GU550727 |  |  |  |
| *Cyrtodactylus petani* | KU232620 |  |  |  |
| *Cyrtodactylus philippinicus* | JX440550 | JX440609 | JX440660 | JQ945304 |
| *Cyrtodactylus phuquocensis* | KT013182 |  |  |  |
| *Cyrtodactylus pronarus* | HQ401163 |  |  |  |
| *Cyrtodactylus pronarus* | HQ401163 |  |  |  |
| *Cyrtodactylus pseudoquadrivirgatus* | KT013184 |  |  |  |
| *Cyrtodactylus pubisulcus* | JX4405510 | JX440610 | JX440661 | JX440710 |
| *Cyrtodactylus pulchellus* |  |  |  | JX440711 |
| *Cyrtodactylus pulchellus* | JX440552 | JX440611 | JX440662 |  |
| *Cyrtodactylus quadrivirgatus* | JX440553 | JX440612 | JX440663 | JX440712 |
| *Cyrtodactylus quadrivirgatus* | KU253580 |  |  |  |
| *Cyrtodactylus redimiculus* | GU550740 |  |  |  |
| *Cyrtodactylus robustus* | JX440554 | JX440613 | JX440664 | JX440713 |
| *Cyrtodactylus russelli* | JX440555 | JX440614 |  | JX440714 |
| *Cyrtodactylus sadlieri* | JQ820309 |  |  |  |
| *Cyrtodactylus sadlieri* | MH105036 |  |  |  |
| *Cyrtodactylus sadlieri* | MH105037 |  |  |  |
| *Cyrtodactylus sadlieri* | MH105038 |  |  |  |
| *Cyrtodactylus sadlieri* | JQ820309 |  |  |  |
| *Cyrtodactylus salomonensis* | JX440556 | JX440615 | JX440665 | JX440715 |
| *Cyrtodactylus semenanjungensis* | JQ889177 | JX440616 | JX440666 | JX440716 |
| *Cyrtodactylus seribuatensis* | JX440557 | JX440617 | JX440667 | JX440717 |
| *Cyrtodactylus sermowaiensis* | JX440558 | JX440618 | JX440668 | JX440718 |
| *Cyrtodactylus serratus* | JQ820297 |  |  |  |
| *Cyrtodactylus sharkeri* | KJ659853 |  |  |  |
| *Cyrtodactylus slowinskii* | JX440559 | JX440619 |  | JX440719 |
| *Cyrtodactylus sp. "Kai Islands"* | MF706380 |  |  |  |
| *Cyrtodactylus sp. "Mt Pekopekowana"* | HQ401193 |  |  |  |
| *Cyrtodactylus sp. "Timor"* | JX440560 | JX440620 | JX440669 | JX440720 |
| *Cyrtodactylus sp. Bali* | KU232624 |  |  |  |
| *Cyrtodactylus sp. Bali* | KU232625 |  |  |  |
| *Cyrtodactylus sp. East Nusa Tenggara, Indonesia* | KU232623 |  |  |  |
| *Cyrtodactylus sp. East Timor* | KU232622 |  |  |  |
| *Cyrtodactylus sp. Yamdena* | KU232621 |  |  |  |
| *Cyrtodactylus tanim* n. sp. | MF706376 |  |  |  |
| *Cyrtodactylus tautbatorum* | GU366083 |  |  |  |
| *Cyrtodactylus tibetanus* | JX440561 |  |  | JX440722 |
| *Cyrtodactylus tigroides* | JX440562 |  | JX440671 | JX440723 |
| *Cyrtodactylus tiomanensis* | JX440563 | JX440622 | JX440672 | JX440724 |
| *Cyrtodactylus trilatofasciatus* | JX519529 |  |  |  |
| *Cyrtodactylus tripartitus* | HQ401203 |  |  |  |
| *Cyrtodactylus tripartitus* | HQ401203 |  |  |  |
| *Cyrtodactylus tuberculatus* | JX440564 |  | JX440673 | JX440725 |
| *Cyrtodactylus tuberculatus* |  | JX440623 |  |  |
| *Cyrtodactylus yangbayensis* | KT013202 |  |  |  |
| *Cyrtodactylus yoshii* | JX440565 | JX440624 | JX440674 | JX440726 |

Table S2. Details of samples of *Lepidodactylus* (and allied genera) included in analyses.

| ***Taxon Name*** | **CATNUM** | **FIELDNUM** | **Tissue Number** | **ND2** | **RAG-1** | **Phosducin** | **Locality** |
| --- | --- | --- | --- | --- | --- | --- | --- |
| *Lepidodactylus aurolineatus* |  | 1687 | ABTC50554 | MG780702 | MG780649 |  | Philippines: Camiquin |
| *Lepidodactylus balioburius* | KU326207 | RMB 7612 |  | MG780708 |  |  | Philippines: Batanes Province, Municipality of Mahatao "Ijang" area, south of Basco town proper |
| *Lepidodactylus christiani* |  | CM311 | ABTC32655 | MG780702 | MG780649 |  | Philippines: Negros Island, Camp Lookout |
| *Lepidodactylus euaensis* | USNM322126 |  |  | JX515611 | JX515628 | JX515641 | Tonga: Eua Island |
| *Lepidodactylus flavocularis* | KU 341207 | SLT 100 |  | MG780716 |  |  | Solomon Islands: Guadacanal, Barana Village |
| *Lepidodactylus guppyi* | USNM533293 |  |  | JX515620 | JX515635 | JX515647 | Solomon Islands: Taumako Island |
| *Lepidodactylus herrei herri* |  | CM285 | ABTC32638 | MG780717 | MG780650 |  | Philippines: Negros Island, Dumaguete City |
| *Lepidodactylus herrei medianus* | PNM 9688 | RMB 4330 |  | MG780 |  |  | Philippines: Leyte Island, Baranday Danao, Albuera |
| *Lepidodactylus listeri* | ABTC50488 |  |  | GQ257746 |  |  | Australia: Christmas Island |
| *Lepidodactylus listeri* | R32507 |  | 14644 | MG780722 | MG780655 |  | Australia: Christmas Island |
| *Lepidodactylus listeri* |  | TT249 | ABTC6881 | MG780725 | MG780657 |  | Australia: Christmas Island |
| *Lepidodactylus listeri* |  | CATALOG6:1 | 6880 | MG780724 | MG780656 |  | Australia: Christmas Island |
| *Lepidodactylus lugubris* |  | SJR14238 | ABTC136590 | MG780726 | MG780658 |  | Papua New Guinea: Manus Island, Piri Village |
| *Lepidodactylus magnus* | SAM |  | ABTC50584 | MG780800 | MG780680 | MG780627 | Papua New Guinea: Madang Province, Kaironk |
| *Lepidodactylus manni* |  |  | ABTC32753 | MG780761 | MG780667 |  | Fiji: Viti Levu |
| *Lepidodactylus moestus* | USNM521730 |  |  | JN019079 | JN019143 | JN019111 | Palau Islands: Ngerur Island |
| *Lepidodactylus orientalis* |  | ENR0175 |  | MG780763 |  |  | Papua New Guinea: Port Moresby; Waigani; National Research Institute (NRI) grounds |
| *Lepidodactylus planicaudus* |  | ACD 1606 |  | MG780773 |  |  | Philippines: Mindanao Island, Mt Apo |
| *Lepidodactylus pumilus* | LSUMZ 97472 | CCA 16142 |  | MG780774 | MG780672 |  | Papua New Guinea: Western Province, Daru Island |
| *Lepidodactylus ranauensis* |  | ID7174 |  | MG780776 |  |  | Malaysia: Sabah, Mt Kinabalu Park Headquarters |
| *Lepidodactylus sp. 1 Bicol* | KU 331652 | RMB 13781 |  | MG780778 |  |  | Philippines: Luzon Island, Municipality of Malinao, Barangay Tanawan |
| *Lepidodactylus sp. 2 N. Luzon* | KU 330065 | RMB 14765 |  | MG780782 |  | MG780609 | Philippines: Municipality of Gonzaga, Barangay Magrafil, Mt. Cagua |
| *Lepidodactylus sp. 3 W. Luzon* | KU 320410 | CDS 3930 |  | MG780784 |  | MG780611 | Philippines: Lubang Island, Occidental Mindoro Province, Sitio Dangay |
| *Lepidodactylus sp. 4. Widespread* | | RMB 5723 |  | MG780789 |  | MG780616 | Philippines: Cagayan Island, Barangay Balatabat |
| *Lepidodactylus sp. 5 Zamboanga* | KU 321505 | RMB 11723 |  | MG780618 | MG780791 |  | Philippines: Mindanao Island, Municipality of Pasonanca Pasonanca Natural Park, Tumaga River |
| *Lepidodactylus sp. Amau* | LSUMZ 95824 | CCA 5700 |  | MG780795 |  | MG780622 | Papua New Guinea: Central Province, Amau River |
| *Lepidodactylus sp. Boiabowaga* | BPBM15842 | FK 5786 |  | JX515617 | JQ945312 | JQ945380 | Papua New Guinea: Milne Bay Province, Boiaboiawaga Is. |
| *Lepidodactylus sp. Bundi* | AMSR124447 |  | ABTC48538 | MG780798 | MG780678 | MG780625 | Papua New Guinea: Madang Province, Bundi |
| *Lepidodactylus sp. Kamiali* | BPBM40272 | AA 20260 | PCMB5992 | MG780807 | MG780684 | MG780631 | Papua New Guinea: Morobe Province, Kamiali |
| *Lepidodactylus sp. Maluku* | TNHC59447 | JAM2248 |  | MG780802 |  |  | Indonesia: Buru Island |
| *Lepidodactylus sp. Misima* | BPBM17229 | FK 6989 |  | MG780808 | MG780685 | MG780632 | Papua New Guinea: Misima Island, Liak |
| *Lepidodactylus sp. New Britain* | SAMAR64666 | SJR10670 | ABTC104666 | MG780809 | MG780686 | MG780633 | Papua New Guinea: West New Britain, Lamas Camp |
| *Lepidodactylus sp. Pacific* |  |  | ABTC50544 | MG780812 | MG780689 |  | French Polynesia: Takapoto |
| *Lepidodactylus sp. Salawati* |  | SJR7808 | ABTC90234 | MG780817 |  | MG780641 | Indonesia: Papua Barat, Salawati Island |
| *Lepidodactylus sp. Sepik* |  | SJR13234 | ABTC114706 | MG780810 | MG780634 | MG780634 | Papua New Guinea: Sanduan Province, Sepik Basin |
| *Lepidodactylus sp. Seribuat* | LSUHC 6899 |  |  | MG780818 |  |  | Malaysia: Seribuat Archipelago |
| *Lepidodactylus sp. Sudest* | BPBM19794 |  |  | JN19080 | JN019144 | JN019112 | Papua New Guinea: Sudest Island |
| *Lepidodactylus sp. Trans-Fly* |  | ENR0706 |  | MG780712 | MG780651 |  | Papua New Guinea: Western Province, Mibini Village, 11 km S of Morehead |
| *Lepidodactylus sp. Umwate* | BPBM42860 | AA 22207 |  | MG780819 | MG780819 | MG780642 | Papua New Guinea: Morobe Province, Umwate |
| *Lepidodactylus sp. Utai* | LSUMZ 95849 | CCA 3475 |  | MG780821 | MG780696 | MG780644 | Papua New Guinea: Sanduan Province, Utai Village |
| *Lepidodactylus sp. Wewak* | LSUMZ 97475 | CCA 16952 |  | MG780823 | MG780697 | MG780644 | Papua New Guinea: Madang Province, Wewak, Kreer Heights |
| *Lepidodactylus sp. Woodlark* | BPBM39152 | FK 14963 |  | MG780824 |  | MG780647 | Papua New Guinea: Woodlark Island, Piak Track |
| *Lepidodactylus sp.6 Bulakan* | deposited in PNM, Uncataloged | ACD 6052 |  | MG780792 |  | MG780619 | Philippines: Luzon Island, Balacan Provimnce, Miguel, Barangay Biak na Bato |
| *Lepidodactylus vanuatuensis* | USNM32547 |  | ABTC50700 | MG780828 | MG780699 | MG780648 | Vanuatu: |
| *Luperosaurus anglit* | KU322189 | ACD 3678 |  | JQ437903 |  | JQ437944 | Philippines: Aurora Province, Aurora State College of Technology |
| *Luperosaurus cf. mcgregori* |  | ACD 6021 |  | JQ437901 |  |  | Philippines: Calayan Island |
| *Luperosaurus cumingii* | TNHC61910?? | RMB3546 |  | JX515623 | JX515637 | JX515650 | Philippines: Cumiaguan Island |
| *Luperosaurus mcgregori* | KU304850 | RMB 5971 |  | JX515624 | JX515638 | JX515651 | Philippines: Cagayan Island |
| *Pseudogekko atiorum* | KU302818 | CDS 954 |  | KF875323 | KF875377 |  | Philippines: Negros Island, Negros Oriental Province, Mt. Talinis |
| *Pseudogekko brevipes* |  | ACD7255 |  | KF875330 |  |  | Philippines: Leyte Island, Municipality of Sogud, |
| *Pseudogekko chavacano* | KU314963 | ACD 3784 |  | KF875339 | KF875392 |  | Philippines: Mindanao Island, Pasonanca Natural Park |
| *Pseudogekko compressicorpus* | KU330058 | RMB 15139 |  | KF875327 | KF875381 |  | Philippines: Luzon Island, Cagayan Province, Mt. Cagua |
| *Pseudogekko ditoy* | KU326437 | RMB 4365 |  | JX515625 | JX515639 | JX515652 | Philippines: Leyte island, Sitio Cienda |
| *Pseudogekko pungkaypinit* | KU324426 | CDS 5000 |  | JQ437898 | JQ437941 |  | Philippines: Bohol Island, Barangay Danicop, Raja Sikatuna Natural Park |
| *Pseudogekko smaragdinus* | KU303995 | RMB 5598 |  | JX515626 | JQ945332 | JQ945401 | Philippines: Luzon Island, Quezon Province, N/A |
| *Gehyra mutilata* | LSHC7376 |  |  | JN393913 | JN393958 | JN393991 | Cambodia: Phnom Aural |
| *Gekko athymus* | KU 309335 |  |  | JN019075 | JN019139 | JN019107 | Philippines: Palawan |
| *Gekko auriverrucosus* | NNU Z20050716.004 |  |  | JN019062 | JN019127 | JN019096 | China: Yuncheng |
| *Gekko badenii* |  |  | JB13 | JN019065 | JN019130 | JN019099 | captive |
| *Gekko carusadensis* | KU328371 |  |  | JQ173404 |  |  | Philippines: Bulacan Province, Luzon Island |
| *Gekko chinensis* | LSHC 4209 |  |  | JN019058 | JN019123 | JN019092 | China: Hainan Island |
| *Gekko coi* | KU326208 |  |  | JN710491 |  |  | Philippines: Sibuyan Island |
| *Gekko crombota* | KU 304830 |  |  | FJ487874 |  |  | Philippines: Babuyan Claro Island |
| *Gekko ernstkelleri* | KU300196 |  |  | JQ173411 | JQ173550 |  | Philippines:Panay |
| *Gekko gecko* | MZB Lace 6628 |  |  | JN019048 | JN019115 | JN019083 | Indonesia: Sumatra |
| *Gekko gigante* | KU302716 |  |  | JQ173417 |  |  | Philippines: Gigante Islands |
| *Gekko grossmani* |  |  | JFBM 9 | JN019064 | JN019129 | JN019098 | Captive |
| *Gekko hokouensis* | NNU Z20050902.001 |  |  | JN019060 | JN019125 | JN019094 | China: Jinzhai |
| *Gekko japonicus* | NNU Z20050801.004 |  |  | JN019059 | JN019124 | JN019093 | China: Zhoushan |
| *Gekko kikuchi* | HOFH89053101 |  |  | KJ018155 |  |  | Taiwan, Lanyu Island |
| *Gekko mindorensis* | KU 303912 |  |  | JN019076 | JN019140 | JN019108 | Philippines: Mindoro |
| *Gekko monarchus* | LSHC 4824 |  |  | JN019078 | JN019142 | JN019110 | Malaysia: Selangor |
| *Gekko petricolus* |  |  | JB70 | JN019066 | JN019131 | JN019100 | captive |
| *Gekko romblon* | KU302738 |  |  | JN710492 |  |  | Philippines: Romblon Island |
| *Gekko rossi* | KU304876 |  |  | FJ487871 |  |  | Philippines: Calayan Island |
| *Gekko smithi* | ID 8774 | ID 8774 | ID 8774 | JN019054 | JN019119 | JN019088 | Malaysia: Sarawak |
| *Gekko sp. Dalupiri Is.* | KU 307054 |  |  | FJ487882 |  |  | Philippines: Dalupiri Island |
| *Gekko subpalmatus* | AMB 6567 |  |  | JN019063 | JN019128 | JN019097 | China: Chengdu |
| *Gekko swinhonis* | NNU Z20051124.001 |  |  | JN019061 | JN019126 | JN019095 | China: Boa |
| *Gekko vittatus* | BPBM19780 |  |  | JN019069 | JN019134 | JN019102 | Papua New Guinea: Rossel Island |
| *Ptychozoon horsfieldi* | ZRC 2.5339 |  |  | JQ437907 | JQ437949 |  | Malaysia: Sarawak, Lambir Hills |
| *Ptychozoon intermedium* | PNM 2501 |  |  | JQ437908 | JQ437950 |  | Philippines: Municipality of Calinan, Barangay Malagos |
| *Ptychozoon kuhli* |  | RMB 1134 |  | JQ437919 | JQ945334 | JQ945403 | Malaysia |
| *Ptychozoon lionatum* | CAS 221168 |  |  | JX515627 | JQ945335 | JQ945404 | Myanmar: Bago Division |
| *Ptychozoon lionatum* | LSHC 6437 |  |  | JQ437914 | JX515640 | JQ437956 | Malaysia: Pahang |
| *Ptychozoon trinotaterra* | ROM 31912 |  |  | JQ437912 | JQ437954 |  | Vietnam: Yok Don National Park |

Table S3. Details of samples and specimens included in analyses for *Cryptoblepharus.*

| **Genus** | **species** | **ID** | **ND2** | **location** |
| --- | --- | --- | --- | --- |
| *Cryptoblepharus* | *adamsi* | QMJ48420/ABTC16251 | MH216007 | Australia: Townsville, Queensland |
| *Cryptoblepharus* | *burdeni* | WAM R104772 | MH216005 | Indonesia: Pasir Island |
| *Cryptoblepharus* | *daedalos* | CCM0619 | MH216021 | Australia: Vic River Region Joe Creek |
| *Cryptoblepharus* | *egeriae* | AMSR R152685 | MH216022 | Australia: Old Mine Fields 25 & Ml139 Nth. Murray Hill |
| *Cryptoblepharus* | *juno* | CCM2973 | MH216019 | Australia: East Baines Camp |
| *Cryptoblepharus* | *juno* | BP02478 | MH216017 | Australia: Berkeley SR |
| *Cryptoblepharus* | *leschenault* | WAM R105231 | MH216004 | Indonesia: Merdeka |
| *Cryptoblepharus* | *megastictus* | NTM R22789 | MH216015 | Australia: Kalumburu |
| *Cryptoblepharus* | *mertensi* | NTM R35690 | MH216009 | Australia: Roper Bar |
| *Cryptoblepharus* | *metallicus* | CCM0390 | MH216018 | Australia: Macarthur River HS |
| *Cryptoblepharus* | *metallicus* | CCM0259 | MH216016 | Australia: Julius dam |
| *Cryptoblepharus* | *nigropunctatus* | 13-5130 | MH216020 | Japan: Ogasawara gunto |
| *Cryptoblepharus* | *novaeguineae* | ABTC90118 | MH216006 | Indonesia: Raja Ampat |
| *Cryptoblepharus* | *novocaledonicus* | AMS R163245 | GBKX231475 | New Caledonia: Ile Aventure, Ile des pins |
| *Cryptoblepharus* | *pannosus* | CCM0071 | MH216008 | Australia: Lornevale |
| *Cryptoblepharus* | *poecilopleurus* | MS082 | MH216013 | French Polynesia: Fakarava - Tetamanu |
| *Cryptoblepharus* | *ruber* | CCM1451 | MH216012 | Australia: Chamberlain valley camp |
| *Cryptoblepharus* | *tytthos* | NMV Z29018 | MH216023 | Australia: Wilare Bridge |
| *Cryptoblepharus* | *virgatus* | CCM5274 | MH216011 | Australia: West of Watsonville, 8.6km E of Irvine Bank |
| *Cryptoblepharus* | *yulensis* | SJR10585/ABTC101989 | MH216014 | Papua New Guinea: Juha survey |
| *Cryptoblepharus* | *zoticus* | CCM0421 | MH216010 | Australia: Massacre Hill Camp site |

Table S4. Details of *Emoia* included in the study. Full details are only given for newly sequenced samples.

| **Tipname** | **Specimen** | | **ND2** | **Locality** | | |
| --- | --- | --- | --- | --- | --- | --- |
| Emoia atrocostata.01 | RNF246 | | MH124073 | Papua New Guinea: Madang, Pig Island | | |
| Emoia atrocostata.07 | USNM576201 | | MH124074 | Federated States of Micronesia: Caroline Islands, Yap Island | | |
| Emoia atrocostata.63 | JQR1889 | | MH124075 | Solomon Islands:Choiseul, Taro Island | | |
| Emoia boettgeri.01 | USNM576213 | | MH124071 | Micronesia, Federated States of, Caroline Islands, Pohnpei Island Kolonia | | |
| Emoia caeruleocauda | LSUMZ93869 | | KU851260 | Papua New Guinea | | |
| Emoia cyanura.04 | K31* | | MH124077 | Micronesia, Federated States of Kosrae, Lele Island, Tradewinds Hotel | | |
| Emoia cyanura.06 | TC1730* | | MH124078 | Republic of Fiji, Taveuni Island | | |
| Emoia impar | USNM558760 | | KU851315 | Micronesia | | |
| Emoia isolata. | RNF757* | | KU851365 | Solomon Islands | | |
| Emoia nativitatis.02 | FS01* | | MH124072 | Christmas Island, Egeria Point | | |
| Emoia ponapea.01 | X3662_pon | | KU851366 | Ponape | | |
| Emoia pseudocyanura | LSUMZ93845 | | KU851368 | Papua New Guinea | | |
| Emoia rufilabialis.01 | USNM533508 | | MH124076 | Solomon Islands Temotu Santa Cruz Island | | |
| Emoia schmidti. | KU307097 | | KU851375 | Solomon Islands | | |
| Emoia taumakoensis. | USNM:533524 | | KU851380 | Solomon Islands | | |
| * USGS Fieldnumbers |  | |  |  | | |
|  | |  | | |  |  |

Table S5. Calibrations and sequence data summary for all taxa

| **Species** | **Analysis** | **Alignment** | **N taxa** | **Calibrations** | **Calibrations source** |
| --- | --- | --- | --- | --- | --- |
| ***Cyrtodactylus sadleiri*** | ***1*** | 1041bp ND2, 278bp tRNA, 836 bp MXCA1, 1050 bp RAG-1, 390 Phos | 129 | *C. angularis*/*C. novaeguineae* - mean 21.0, stdev 4.0 *C.ayeyawardyensis*/*C.novaeguineae* - mean 31.0, stdev 5.0 | Secondary cals from analysis of 5 nuc genes presented in Oliver et al. 2017 (4 fossil calibrations, BD model, lgnm prior preferred) |
|  | 2 | as above, 3rds and RNA excluded | 129 |  |  |
|  |  |  |  |  |  |
| ***Lepidodactylus listeri*** | ***1*** | 1041bp ND2, 1035 bp RAG-1, 395bp Phos | 53 | Crown Gekko - mean 36.0, st.dev 4.0 *Lepidodactylus* - mean 34.0, st.dev 4.0 | Secondary cals from analysis of 5 nuc genes presented in Oliver et al. 2017 (4 fossil calibrations, BD model, lgnm prior preferred) |
|  | *2* | as above, 3rds and tRNA excluded | 53 | as above |  |
|  | *3* | RAG-1, Phos | 29 | as above |  |
|  |  |  |  |  |  |
| ***Emoia nativitatis*** | ***1*** | 1041bp ND2 | 15 | node - *Emoia cyanura* to *E.impar* (uniform 10.5-14.5)  rate - 1-4% pairwise, uniform | Secondary cals for other sampled taxa of Emoia + broad prior on overall molecular rate (see SI methods) |
|  |  |  |  |  |  |
| ***Cryptoblepharus egeriae*** | ***1*** | 1173bp ND2 | 21 | node - *C nigropunctatus* v *novocaledonicus* (uniform 3.5-5.5mya)  rate - 1-4% pairwise, uniform | Secondary cals for other sampled taxa of *Cryptoblepharus* + broad prior on overall molecular rate (see SI mthods) |

Figure S1. Nominal boundaries of the Sunda, Wallacean and Australian biotic regions, in relation to Christmas Island (demarcated by a red star).


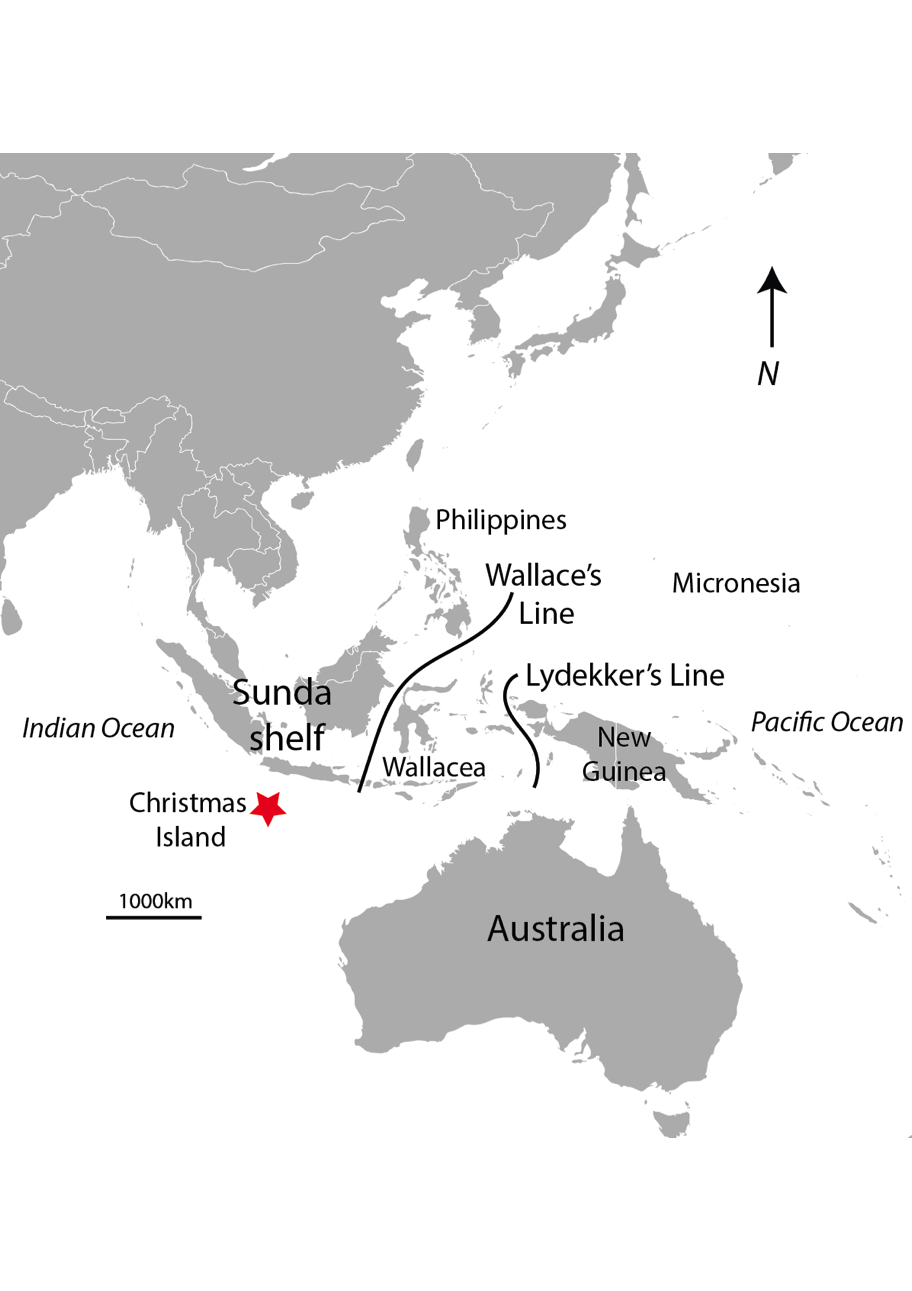


Figure S2. Chronogram for gekkotans estimated using a five nuclear gene dataset and 4 primary fossil calibrations largely from Gamble et al. 2015. Estimated timeframe for evolution within the focal gecko genera are shown in detail with key nodes used to calibrate downstream analyses highlighted. Timescale of lower axes in millions of years ago.

Figure S3 Chronogram for *Cyrtodactylus* estimated from combined mitochondrial and nuclear data and two secondary node priors. Timescale of lower axis in millions of years ago. Only tree with highest marginal likelihood is shown (lognormal and Yule speciation prior). Node bars indicate 95% HPD distribution for node ages, Bayesian posterior supports shown at key nodes. Colour branches respectively demarcate taxa from Christmas Island (red), and related taxa from Wallacea (blue) and the Sunda or Australian continental shelfs (green).

Figure S4. Chronogram for *Lepidodactylus* and allied genera estimated from a) combined mitochrondrial and nuclear data and b) nuclear data only. Timescale of lower axis in millions of years ago. Coloured branches respectively demarcate taxa from Christmas Island (red), and inferred relatives from the Philippines (blue). Note lower tree has fewer samples, as nuclear data was only available for a subset of taxa.

Figure S5. Chronogram for *Eugongylus* group skinks estimated using topology extracted from Pyron et al. 2013, calibrated using penalised rate smoothing and 4 mean age priors taken from Skinner et al. 2011. Timescale of lower axis in millions of years ago. Variation in age estimates (node bars) generated by varying rate smoothing parameter from 0.5-20.0.


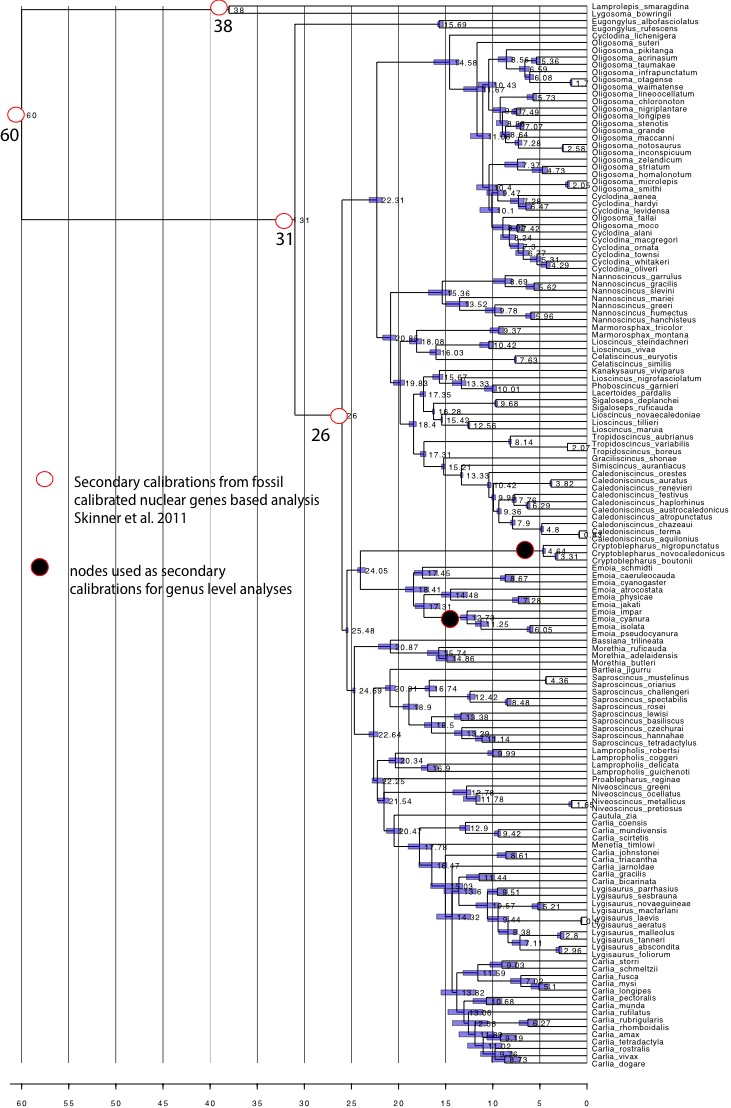


Figure S6. Chronogram for *Cryptoblepharu*s estimated from mitochondrial ND2 data and combined secondary and molecular rate priors. Only tree with highest marginal likelihood is shown (strict-clock and Yule speciation prior). Timescale of lower axis in millions of years ago. Node bars indicate 95% HPD distribution for node ages. Bayesian posterior supports shown at key nodes. Coloured branches respectively demarcate taxa from Christmas Island (red), Australia (blue) and Lesser Sundas (green).

Figure S7. Chronogram for *Emoia* estimated from mitochondrial ND2 data and combined secondary and molecular rate priors. Only tree with highest marginal likelihood is shown (strict-clock and Yule speciation prior). Timescale of lower axis in millions of years ago. Node bars indicate 95% HPD distribution for node ages. Bayesian posterior supports shown at key nodes. Colour branches respectively demarcate taxa from Christmas Island (red) and its putative closest relative from Micronesia (blue), and one distantly related taxon also known from the Lesser Sundas (green).
